# RNAiSeq: How to See the Big Picture

**DOI:** 10.3389/fmicb.2019.02570

**Published:** 2019-11-14

**Authors:** Brenda Oppert, Lindsey Perkin

**Affiliations:** ^1^USDA, Agricultural Research Service, Center for Grain and Animal Health Research, Manhattan, KS, United States; ^2^USDA, Agricultural Research Service, Southern Plains Agricultural Research Center, College Station, TX, United States

**Keywords:** gene expression, RNASeq, stored product insect, topical RNAi, *Tribolium castaneum*, pest control product

## Abstract

Targeting genes *via* RNA interference (RNAi) has become a successful method to reduce pest populations. Ideally, the expression of a gene critical for a life function in the insect is targeted by specific dsRNA, *via* spray or oral delivery. Experts have developed working guidelines in the development and regulation of RNAi as a pesticide. We argue that an important tool in the validation of RNAi is genome-wide expression analysis in the targeted pest, and we name this approach RNAiSeq. We have used RNAiSeq in the coleopteran model *Tribolium castaneum* to validate knockdown of target genes, and to examine the effect of knockdown on other genes. With RNAiSeq, we identified compensation responses to the knockdown of a gene encoding a major digestive enzyme in larvae that correlated to the responses we have observed with ingested protease inhibitors. Compensation can mask RNAi phenotypic responses and is important to understand in the context of efficacy. RNAiSeq also has identified new gene interactions that were previously unassociated with the target gene, important in the context of the large number of genes without associated functions in insects and other organisms. We discuss other research where RNAiSeq has led to important findings. These data not only provide validation of target knockdown, but also further identify changes in the expression of other genes impacted by the knockdown. From the context of pest control, this information can be used to predict genetic changes that will impact the efficacy of RNAi products in target pests.

## Introduction

RNA interference (RNAi) is one of the mostly widely used tools to study gene function in insects. RNAi is also a potential control product being developed to combat problem pests *via* either oral or topical application. We and others have discussed the benefits and problems associated with RNAi (Baum et al., 2007; Noh et al., 2015; Joga et al., 2016; Perkin et al., 2016b). Our research has focused on developing new insect control products, including RNAi, for stored product beetles.

**Figure 1:**
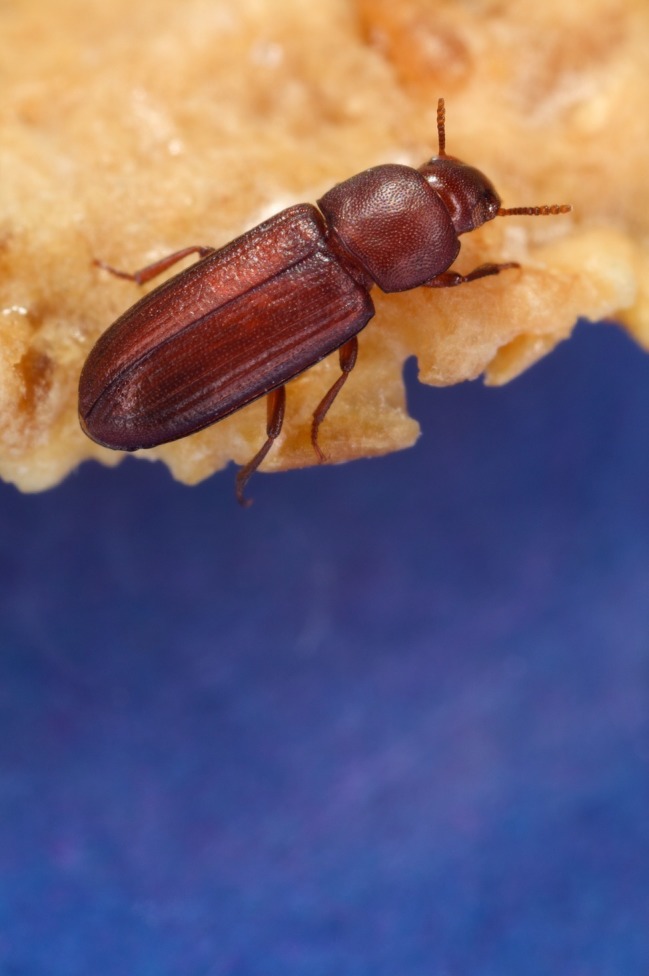
*Tribolium castaneum* adult feeding on grain (photo courtesy USDA ARS, Peggy Greb).

Our model for the development of RNAi for stored product pest control is the red flour beetle, *Tribolium castaneum* (Figure 1). This insect feeds on grains and stored products and inflicts major economic damage worldwide (Pimentel, 1991). Flour beetles are responsible for losses in stored grains, warehouses, and flour mills, among others. Fumigants such as phosphine are the most common control product, but many populations of phosphine-resistant beetles and other stored product insects have been identified (Pimentel et al., 2010; Opit et al., 2012).

*T. castaneum* has served as a genetic model for coleopteran research and was the first agriculturally important insect to have a sequenced genome (Tribolium Genome Sequencing Consortium, 2008). *T. castaneum* has a robust response to injected RNAi (Brown et al., 1999; Tomoyasu and Denell, 2004; Aronstein et al., 2011; Miller et al., 2012), but success with oral RNAi has been documented in only a few studies (Whyard et al., 2009; Cao et al., 2018). We and others have not observed a phenotype or mortality response to oral RNAi and have postulated that the problem is either nucleases in the alimentary canal, or lack of transport in the gut (Palli, 2014, unpublished data).

Here we discuss the value of using RNA-Seq as a validation tool for RNAi, which we refer to as RNAiSeq, and we demonstrate the value of RNAiSeq in case studies from our research. We also demonstrate how RNAiSeq has been used in other organisms to make important discoveries.

## Case Studies

### Our Case Studies

**Figure 2:**
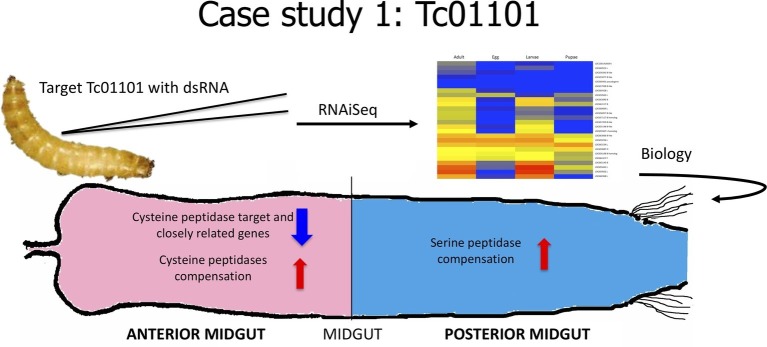
Cartoon depicting analysis from Case Study 1, data from Perkin et al., 2017a.

In our first case study (Figure 2), we sought to understand the effect of knockdown of a major digestive enzyme. Cysteine peptidases are major digestive enzymes in the anterior midgut of *T. castaneum*, but they also have other physiological roles in the insect (Vinokurov et al., 2009; Perkin et al., 2016a). In the functional characterization of cysteine peptidases in *T. castaneum*, we found that one gene, *TC01101*, encoded the primary cysteine peptidase and was the mostly highly expressed gene in the larval gut (Morris et al., 2009; Perkin et al., 2016a). We used RNAiSeq to investigate the effect of knockdown of *TC01101* using dsRNA designed from the 3’ end, 5’ end, middle, and entire sequence (Perkin et al., 2017a). All constructs resulted in significant reduction in *TC01101* gene expression. However, other cysteine peptidase genes were increased in expression, effectively compensating for the loss of *TC01101* and masking any loss-of-function effects of the target gene. This compensation response also was accompanied by increased expression of serine peptidase genes. Importantly, these responses paralleled the compensation we had observed at the protein level in insects fed protease inhibitors (Oppert et al., 1993, 2005, 2010). The data provided crucial molecular evidence to explain how insects adapt to and survive on diets containing protease inhibitors through the regulation of an intricate network of duplicated genes. The remaining piece of the puzzle is to identify the regulatory elements responsible for the adjustments in gene expression to compensate for inhibitors or other disruptive dietary compounds (such as dsRNA), research in progress. However, the data demonstrate an evolved and elegant feedback mechanism to conserve digestive efficiency in this stored product beetle.

**Figure 3:**
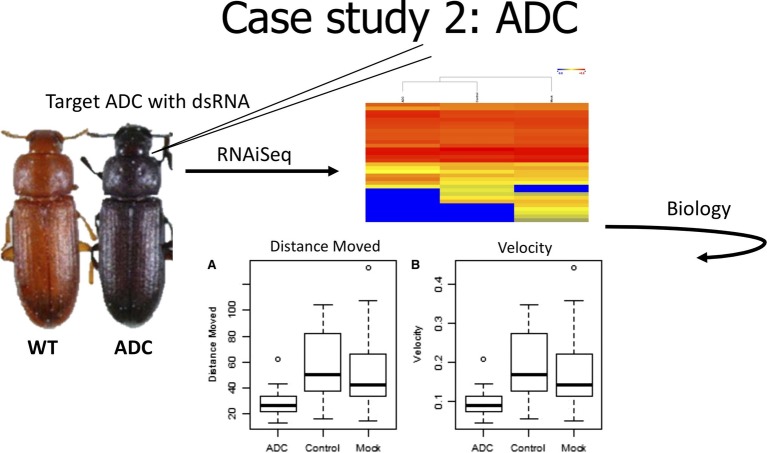
Cartoon depicting analysis from Case Study 2, data from Perkin et al., 2017b.

In the second case study (Figure 3), we evaluated the effect of knockdown of a gene used as a positive phenotypic control. In our early experiments with *T. castaneum*, we commonly used a positive injection control, *aspartate 1-decarboxylase* (*ADC*), because it provided a visual confirmation of knockdown. *ADC* is involved in the cuticle tanning pathway that produces a red phenotype (Arakane et al., 2009), and reduced expression of *ADC* results in a black beetle. RNAiSeq validated the significant knockdown of *ADC*, but it also uncovered a change in the expression of other metabolic genes (Perkin et al., 2017b). These changes included decreased expression of odorant receptors and allatotropin genes, but highly increased expression of *dopamine receptor 2*. In *Drosophila melanogaster*, the increased expression of a dopamine receptor was linked to reduced mobility (Phillips et al., 2005). Therefore, we hypothesized that beetles injected with *ADC-*dsRNA would have slower movement due to increased expression of *dopamine receptor 2.* In fact, dsRNA-injected beetles moved slower and over shorter distances than noninjected control beetles. Therefore, RNAiSeq provided insight into a previously unknown interconnected pathway between *ADC* and *dopamine receptor 2.*

**Figure 4:**
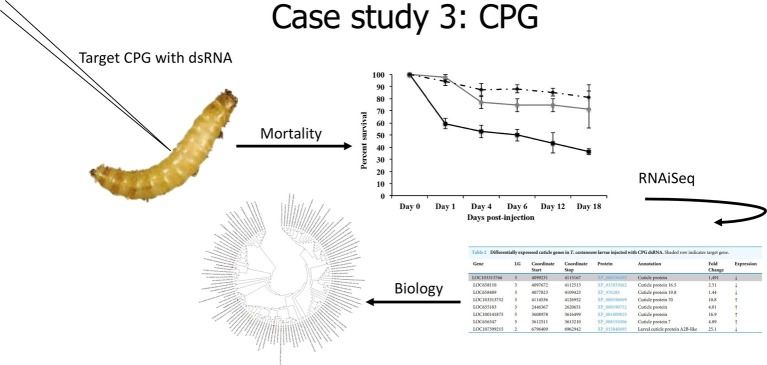
Cartoon depicting analysis from Case Study 3, data from Perkin and Oppert (2019).

In our last case study (Figure 4), we evaluated the effects of the knockdown of a unique cuticle protein gene (*CPG*) found in larvae. Knockdown of *CPG* in *T. castaneum* larvae resulted in significant mortality, and RNAiSeq validated target knockdown as well as the discovery of compensation responses of other cuticle protein genes (Perkin and Oppert, 2019). Interestingly, *CPG* knockdown in *T. castaneum* also induced significant (*p* < 0.01) differential expression of 52 long noncoding RNAs (lncRNAs). Because lncRNA can induce epigenetic changes that alter gene transcription, including silencing (Tufarelli et al., 2003), we propose that these lncRNAs may be involved in the altered expression of *CPG* and related genes. The role of lncRNA in gene silencing mechanisms warrants further research.

**Figure 5:**
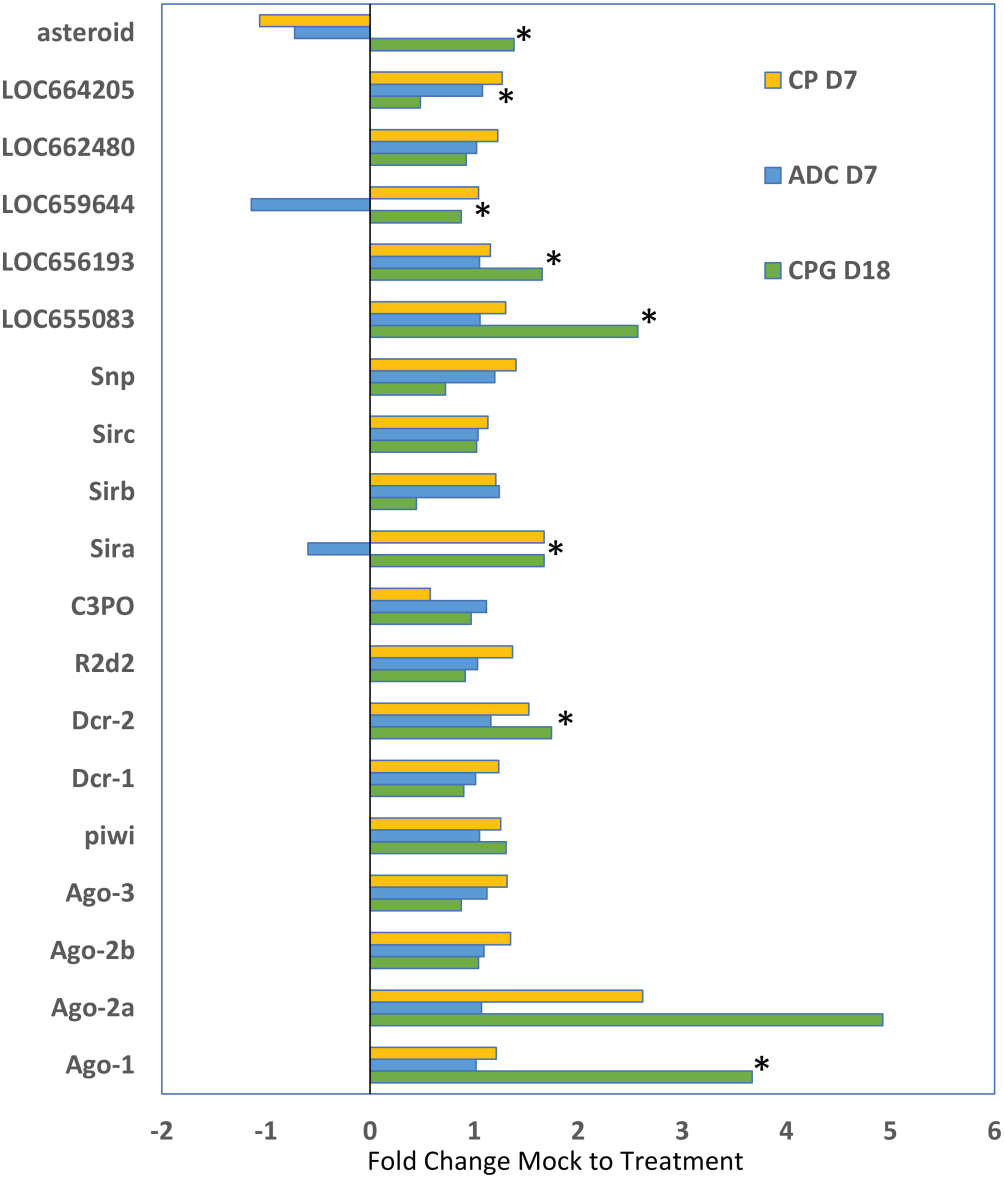
Differential expression of genes typically involved in RNAi (identified on the y axis) as determined by the fold change difference of treatment expression versus that of the control mock injected. CP D7, data from the cysteine peptidase study, analyzed at day 7 post injection (Perkin et al., 2017a); ADC D7 data were from Perkin et al. (2017b) analyzed at day 7 post injection; CPG D18 data were from Perkin and Oppert (2019) analyzed at day 18 post injection. Data that were significantly different (*p* < 0.05) indicated by asterisk.

We compared the expression of genes that are typically associated with RNAi in other insects from these three studies to determine if patterns could be observed in response to injected dsRNA in *T. castaneum* (Figure 5). While only some of the comparisons were statistically significant, the overall trend was that most of the RNAi genes were increased in expression in larvae that were injected with dsRNA. The greatest increase in expression was observed with Ago-1 and Ago-2a (up to 5-fold increase) in the CPG study. The data may reflect the upregulation of RNAi systems in the cell, but more research is needed to determine the mechanisms of increased expression and implications for gene silencing.

### Other Case Studies

An important discovery in understanding the conservation of DNA methylation in eukaryotes was made by RNAiSeq in honeybees. Previously, it was demonstrated that RNAi silencing of *DNA methyltransferase 3* (*dnmt3*) increased the number of worker larvae developing into queens (Kucharski et al., 2008). By combining a unique delivery method for dsRNA (aerosol application through the trachea) and RNASeq and software to identify alternative splicing (Li et al., 2013), four different types of splicing events were detected in response to *dnmt3* gene knockdown (Li-Byarlay et al., 2013). Both exon skipping and intron retention were correlated to decreased methylation from a loss of *dnmt3* function. The data convincingly supported the hypothesis that DNA methylation of genes can regulate alternative splicing, an important finding on how environmental cues can affect gene expression.

In another study, loss of function in cultured cells by either RNAi, antisense oligonucleotides, or CRISPR genome editing of lncRNA or protein coding genes was compared *via* RNAiSeq (Stojic et al., 2018). All methods effectively reduced transcription of the target, but they also induced off target effects. Notably, all methods resulted in differences in both molecular and cellular phenotypes. It was recommended that multiple targeting sequences be evaluated by RNASeq with proper controls that are also compared to untreated samples. Recommendations also included titrating the amount of product (i.e., dsRNA) to use the minimal dose required for a response to reduce off target effects, but this has not been evaluated empirically and may not be practical for the development of products for insect control.

## Discussion

Our experience with combining RNAi and RNASeq, which we now refer to as RNAiSeq, accomplishes the primary task of verification of knockdown of the target gene, but also has yielded unexpected discoveries of gene function and metabolic interactions. We propose that incorporating RNAiSeq into the development of new insect control products, including topical RNAi, provides valuable insights into the response of the transcriptome due to the loss of function from the target gene. This information can be used to increase the potency of the dsRNA by adjusting the dose, choosing a different region of the target DNA, or adjusting the experimental design to avoid reduced product efficacy through compensation responses.

The differential expression of genes other than the target genes is likely not due to direct degradation of transcripts. In most cases, we suspect that the loss of the gene product provides a regulatory response that either decreases or increases the expression of other genes functioning in a network. Exceptions in our studies were likely found in some of the compensation responses of genes that were highly homologous through gene duplication, including genes encoding cysteine proteases or cuticle proteins. Although we sought regions that were unique in the primer design for our dsRNA constructs, it is possible that smaller siRNAs from DICER may have directly interacted with nontarget genes. Bioinformatics is also highly dependent on the accuracy of predicted gene sequences.

Selection of the timepoint(s) for RNAiSeq may need to be verified experimentally. We routinely use day 7 post injection for extraction of RNA, but we extended the timepoint to day 18 in the CPG study (Perkin and Oppert, 2019) due to delayed effects on mortality. Additionally, we used whole larvae in our experiments to observe global transcriptome responses in the whole animal, but RNAiSeq could be used for selected tissues or even single-cell transcriptomics, provided the amount of material is sufficient for libraries. Of course, other techniques, such as proteomics, will provide even more supplemental information on the effects of gene knockdown.

We anticipate that the routine use of RNAiSeq will yield additional important benefits. Wood et al. (2019) posed the exciting paradox that while most of the functional discoveries in yeast proteins were made during the 1990’s, we still have little knowledge of the function of 20% of protein coding genes. Orthologs with unknown function are maintained in the genomes of other organisms, including insects, but what are these highly conserved mystery gene products doing? Domains of unknown function (DUFs) are increasingly implicated in niche roles, such as the discovery of DUF1220 copy number in the severity of autism (Davis et al., 2019). Discovery of protein functions unique to insects also are important in understanding the biology of the organism and crucial to pest control. Alternatively, these unique proteins are finding novel applications, such as the incorporation of spider silk proteins into industrial products (Römer and Scheibel, 2008).

Our studies demonstrate that even with genes that have well-defined functions, additional functions can be identified through observation of transcriptome responses to target gene loss of function or reduced expression. Many genes function in intricate networks, often multiple networks, and defining interconnections in networks can lead to more accurate predictions in disrupting gene function. Ultimately, accurate mapping of these networks will lead to a better understanding of the biology of the organism and provide better tools to combat pests and disease.

## Author Contributions

BO and LP were involved in all aspects of the case studies and also contributed to the presentation and this manuscript.

### Disclaimer

The opinions expressed and arguments employed in this publication are the sole responsibility of the authors and do not necessarily reflect those of the OECD or of the governments of its member countries.

Mention of trade names or commercial products in this publication is solely for the purpose of providing specific information and does not imply recommendation or endorsement by the U.S. Department of Agriculture. The USDA is an equal opportunity employer.

### Conflict of Interest

The authors declare that the research was conducted in the absence of any commercial or financial relationships that could be construed as a potential conflict of interest.
